# Evaluation of an Interprofessional Educational Intervention in Mental Health and Intellectual and Developmental Disability for Health and Social Service Trainees

**DOI:** 10.1007/s40596-024-02063-w

**Published:** 2024-11-20

**Authors:** Gabriel Tarzi, Anupam Thakur, Nicole Bobbette, Megan Pilatzke, Gill Lefkowitz, Kendra Thomson, Alicia Thatcher, Syeda Hasan, Adeen Fogle, Marissa Blake, Ann Hines, Yona Lunsky

**Affiliations:** 1 Azrieli Adult Neurodevelopmental Centre, Centre for Addiction and Mental Health, Toronto, Ontario Canada; 2 Temerty Faculty of Medicine, University of Toronto, Toronto, Ontario Canada; 3 Queen’s University, Kingston, Ontario Canada; 4 Brock University, St. Catharines, Ontario Canada; 5 University of Saskatchewan, Regina, Saskatchewan Canada; 6 Fleming College, Peterborough, Ontario Canada

**Keywords:** Mental health, Medical education, Interprofessional education, Intellectual and developmental disability, Project ECHO

## Abstract

**Objective:**

Adults with intellectual and developmental disabilities (IDD) experience high rates of poor mental health and challenges accessing timely and high-quality services. There is limited interprofessional training on mental health care for this population.

**Methods:**

A virtual, synchronous program based on the Project Extension for Community Healthcare Outcomes (ECHO) Ontario IDD Mental Health program was developed for health and social service trainees. Participants represented 10 disciplines across 12 Canadian university or college programs. The program was taught by a team of health and social service providers together with individuals with lived experience and included didactics and case-based discussions. Program evaluation utilized a pre-, post-, and 12-week follow-up survey design with feedback surveys following each session.

**Results:**

Fifty participants registered for the program; 34 (68%) completed baseline measures and attended at least two sessions. Overall, participants reported high session satisfaction (average rating of 4.47 of 5). Participants demonstrated significant improvement in self-efficacy regarding communication (*p* < 0.001), management of mental health needs (*p* < 0.001), and working across systems (*p* < 0.001). Participants self-reported feeling more knowledgeable about common comorbidities (*p* < 0.001), assessing behavioral challenges (*p* < 0.001), the role of interdisciplinary professionals (*p* < 0.001), and community resources (*p* < 0.001). Improvements were maintained at follow-up across outcomes.

**Conclusion:**

The pilot Project ECHO for health and social service trainees in adult IDD mental health demonstrated high participant satisfaction and positive impact on trainees’ self-efficacy and knowledge. Interprofessional educational interventions can be effectively implemented using virtual technology to teach about other mental health populations requiring multisector care.

For adults with intellectual and developmental disabilities (IDD) and mental health concerns, mental health service needs are often not adequately met [1, 2]. Barriers include limited training that health care providers receive on the mental health needs of adults with IDD and health and social services often being uncoupled from one another resulting in inadequate assessment, treatment, and disjointed care [3–5]. As a consequence, adults with IDD have disproportionate mental health morbidity and health care use [5].

To address these challenges, interprofessional mental health care for adults with IDD has been recommended [6]. The Core Competencies on Disability for Health Care Education outlines interprofessional team-based health care as a core competency and highlights the importance of integrating interprofessional competencies in health care education [7]. One particular interprofessional collaboration and education model, the Project Extension for Community Healthcare Options (ECHO), has been shown to be effective at increasing participants’ capacity to deal with various challenges in patient-care across disciplines and sectors [8].

Utilizing a “Hub and Spoke” model where the Hub is composed of an interprofessional team of collaborators and the Spokes are made up of community-based health service providers, learners engage in didactic and case-based learning to create a community of practice where “all teach, all learn” [9]. Research applying the ECHO model for providers caring for adults with IDD and mental health concerns has demonstrated high participant satisfaction, increased provider self-efficacy, fostered interprofessional collaboration, and facilitated greater cross-sector understanding [10–12].

However, the ECHO model is primarily targeted towards continuing practice development and practitioners, which is a missed opportunity given that trainees, as future practitioners, have these same learning needs. Only one study has applied Project ECHO to trainees exclusively, demonstrating its effectiveness in a small cohort of medical students and internal medicine residents learning about mental health not specific to IDD [13].

Across health and social service disciplines, research has called for further education on caring for individuals with IDD as many trainees do not feel equipped to care for this population [14, 15]. However, to our knowledge, there have been no evaluations of interprofessional education specific to mental health and IDD for trainees that have been published. As such, we designed, implemented, and evaluated a virtual pilot Project ECHO program for health and social service trainees, focused on mental health in adults with IDD.

## Methods

The trainee ECHO was a condensed 4-week program based on content from a previous mental health ECHO for Adults with IDD offered to health and service providers [10, 16]. The program and session topics were based on IDD health and social service competency guidelines, interprofessional competency guidelines, known gaps in health and social service research, and input from individuals with lived experience. The four sessions’ topics were “Supporting the Health of Adults with Developmental Disabilities, Team Based Trauma-Informed Care, Promoting Physical and Mental Health, and Emergencies, Advocating and Embracing Uncertainty.” The Hub team, all with prior ECHO experience included a/an psychiatrist, family physician, nurse, psychologist, occupational therapist, behavior analyst, social worker, parents, siblings, and adults with intellectual disability or autism.

The trainee ECHO program was offered synchronously and virtually over 4 weeks in June 2023. Each session was 90 min with a 5-min introduction, 40 min of didactic teaching, a 40-min anonymized case-based discussion presented by a Hub member or trainee, and a 5-min conclusion. After presenting the case, Hub members and trainees asked clarifying questions then provided recommendations for care management.

Trainees across various health and social service disciplines from across Canada were invited to participate in the program, recruited through social media, student associations, and faculty tied to the ECHO program.

A total of 50 trainees consented to participate. Of these, two individuals did not complete any of the assessment measures and 14 were excluded from the evaluation because they attended 0 sessions (*n* = 7) or 1 session (*n* = 7) only. Those who attended fewer than 2 sessions (*n* = 14) did not differ demographically or on baseline measures from those included in the final sample. Participants cited scheduling conflicts during work hours, health issues, vacation, and time zone differences as factors that impacted their ability to attend.

Participants completed pre-program, post-program, and 12-week follow-up questionnaires, as well as weekly satisfaction surveys. Eligible participants were included in the evaluation of the program if they attended two or more sessions and completed the pre-program and post-program questionnaires.

The pre-program, post-program, and follow-up questionnaires consisted of questions surrounding trainees’ self-efficacy in caring for the physical and mental health for individuals with IDD, self-reported knowledge surrounding care for individuals with IDD, and demographic data. The post-program and follow-up questionnaires had additional items on program feedback and changes in practice. The weekly satisfaction surveys consisted of questions about the session content and teaching format, changes to practice, and general suggestions to improve the sessions. The evaluation of the trainee ECHO is based on Moore’s expanded outcomes framework for assessing learners’ outcomes in educational activities [17].

Participants’ pre-program and post-program questionnaires were matched based on participant ID and were analyzed using paired *t*-tests or Wilcoxon signed-rank tests, and an additional one-way repeated measures ANOVA for follow-up. A significance level of *p* < 0.05 was used for all analyses. Directed content analysis of open-response sections of the surveys, organized by question, was conducted [18]. This study was approved by the Institutional Research Ethics Board and data were collected using REDCap.

## Results

The 34 trainees included in the evaluation of the program, of which 30 also completed the follow-up questionnaire, came from 12 different post-secondary institutions across Canada, with 35.2% in social services programs and 64.7% in health programs. Participants included trainees from medicine, nursing, public health, occupational sciences, rehabilitation sciences, social work, psychology, applied behavior analysis, developmental services, and child and youth services. The average age of participants was 26.4 years old, and a majority identified as women (*n* = 28, 82.4%). The average attendance per session was 28 participants (range, 22 to 37 participants/session).

A total of 93 weekly satisfaction surveys were completed with an average of 23 surveys completed per session (range, 19 to 34). The mean overall satisfaction score across all sessions was 4.47 (out of 5) and ranged from 4.37 to 4.60 for individual sessions. Trainees appreciated being able to participate through the chat function or audio. After the program, 88.2% (*n* = 30) of participants indicated that their expectations for participating in the virtual health program were met.

Participants’ average self-efficacy scores at pre and post demonstrated statistically significant increases in all domains after participation (*p* < 0.05), which were maintained at follow-up (Table 1).

**Table 1:** Mean self-efficacy scores of participants regarding providing care for individuals with IDD at three timepoints

Self-efficacy statements *I am confident in my ability to…*	Mean pre-program score	Mean post-program score	Mean follow-up score	*p* value*
Communicate effectively and prepare for person and family-centered care for adults with intellectual and/or developmental disabilities (IDD)	58.6 (*n* = 34) ————— 60.6 (*n* = 30)	81.0 (*n* = 34) ————— 81.4 (*n* = 30)	————— 78.5 (*n* = 30)	*t*_33_ = 7.03, *p* < 0.001 ————— *F*(2, 87) = 28.8, * p* < 0.00001
Support and manage the mental health of individuals with or suspected of having IDD	52.3 (*n* = 34) ————— 54.3 (*n* = 30)	73.7 (*n* = 34) ————— 74.5 (*n* = 30)	————— 72.7 (*n* = 30)	*t*_33_ = 7.63, *p* < 0.001 ————— *F*(2, 87) = 33.6, *p* < 0.00001
Support and manage the physical health needs of individuals with IDD	48.8 (*n* = 34) ————— 50.6 (*n* = 30)	69.4 (*n* = 34) ————— 69.9 (*n* = 30)	————— 70.6 (*n* = 30)	*t*_33_ = 5.77, *p* < 0.001 ————— *F*(2, 87) = 24.1, *p* < 0.00001
Appropriately manage burnout and build resilience in myself, other health care providers, and caregivers	55.2 (*n* = 34) ————— 58.3 (*n* = 30)	71.4 (*n* = 34) ————— 72.8 (*n* = 30)	————— 75.6 (*n* = 30)	*t*_33_ = 4.98, *p* < 0.001 ————— *F*(2, 87) = 18.1, *p* < 0.00001
Work effectively across health and social systems	64.2 (*n* = 34) ————— 64.0 (*n* = 30)	75.9 (*n* = 34) ————— 76.1 (*n* = 30)	————— 80.1 (*n* = 30)	*t*_33_ = 3.52, *p* = 0.001 *—*———— *F*(2, 87) = 11.3, *p* = 0.00007

*Pre-program and post-program values were analyzed with paired t-test and repeated measurements ANOVA for follow-up

Participants reported statistically significant increases in agreement with knowledge statements at post- versus pre-program (*p* < 0.05), with the increases maintained at follow-up (Table 2).

**Table 2:** Participants’ responses to knowledge questions regarding providing care for individuals with IDD at three timepoints

Knowledge statement	Pre-program	Post-program	Follow-up	*p* value
*When caring for a patient with IDD I feel knowledgeable about…*	% “agree” or “strongly agree”			
Common comorbidities and care issues affecting patients with IDD	55.9% (*n* = 34) —————— 53.3% (*n* = 30)	94.1% (*n* = 34) —————— 93.3% (*n* = 30)	——— 96.7% (*n* = 30)	*Z* = − 3.84, *p* < 0.001
How to assess someone who presents with distressed behaviors/behaviors that challenge	41.1% (*n* = 34) —————— 43.3% (*n* = 30)	85.3% (*n* = 34) —————— 83.3% (*n* = 30)	——— 70.0% (*n* = 30)	*Z* = − 4.11, *p* < 0.001
The role that different disciplines can play in supporting mental health	64.7% (*n* = 34) —————— 66.7% (*n* = 30)	88.3% (*n* = 34) —————— 90.0% (*n* = 30)	——— 93.3% (*n* = 30)	*Z* = − 4.11, *p* < 0.001
Other community resources for people with IDD (day programs, community agencies, advocacy groups, developmental services organizations, etc.)	41.2% (*n* = 34) —————— 46.7% (*n* = 30)	85.3% (*n* = 34) —————— 83.3% (*n* = 30)	——— 83.3% (*n* = 30)	*Z* = − 4.11, *p* < 0.001

When asked if participation in this virtual program will impact their future practice, 67.6% (*n* = 23) of participants responded “yes”, 29.4% (*n* = 10) “no”, and 2.9% (*n* = 1) did not respond. Main themes in impacting future practice included the use of the Health, Environment, Life experiences, and Psychiatric (HELP) diagnosis approach to assess behaviors that challenge newfound clinical resources, patient-centered care approaches, and increased confidence in providing care to an individual with IDD.

Participants were asked to provide general program feedback, feedback on the unique aspects of this program, and their perspectives on the “All teach, all learn” teaching philosophy of ECHO. Regarding their learning, many trainees found that the case-based approach (91.2%, *n* = 31) and having interprofessional health and social service providers (100%, *n* = 34) consolidated learning. Having family members (91.2%, *n* = 31) and self-advocates (94.1%, *n* = 32) as part of the Hub team enhanced their learning. They felt supported and part of a virtual community of practice (91.2%, *n* = 31) and they appreciated the opportunity to share strategies with this community (97.1%, *n* = 33).

Analysis of open text responses following the program identified many factors that trainees attributed to their positive experience. These factors were organized into three overarching themes: “All teach, all learn”—a valuable approach, interprofessional teaching is key to success, and a means to bridge knowledge gaps.

A majority of trainees reported that the “all teach, all learn” philosophy was valuable to their learning and to the overall learning environment. Trainees noted that distributing the responsibility of teaching and learning to all participants created a learning environment that was less hierarchical and intimidating. As one trainee said, this philosophy “promotes a sense of safety in allowing everyone to feel as if they have something valuable to share with others.” The involvement of individuals with lived experience, such as self-advocates and family caregivers, provided trainees with first-hand accounts of navigating both health and social systems. This was novel to trainees and provided valuable insight into the experiences of caregivers and individuals with IDD that trainees emphasized and indicated they would like to see more often in their education.

All trainees indicated that the interprofessional nature of the program was a key aspect of the teaching and their learning by broadening their perspectives beyond a siloed, uni-professional practice lens. With participation from individuals from across different disciplines and sectors, trainees were able to learn how their colleagues in different disciplines could contribute to a case, as reiterated by one trainee, “hearing the questions and suggestions from the inter-disciplinary team brought up thoughts that I had never considered before that I could bring into my own practice.” In addition, having diverse perspectives allowed trainees to broaden their understanding of the teaching content, issues at hand, and the roles of their colleagues to provide more holistic care to individuals with IDD.

Trainees also reported that this program addressed a critical knowledge gap regarding adults with IDD in trainees’ respective curricula. A vast majority of trainees stated that they received little to no training on caring for individuals with IDD, with respect to mental health or otherwise, and recognized the importance of being equipped to provide patient and family-centered mental health care to individuals with IDD. As summarized by one trainee, “there is a definitive lack of discussion on IDD and disability support in general within the medical curriculum and what does exist is often not very patient-centred.” Interestingly, some trainees noted that the knowledge they gained in this program could be applied to other patient populations (e.g., older adults, children with medical complexity).

## Discussion

The pilot Project ECHO in IDD and mental health for health and social service trainees demonstrated high satisfaction from—and impact on—participants. Overall, participants provided positive feedback regarding session content and reported significant improvements in self-efficacy and self-reported knowledge when caring for adults with IDD, particularly in mental health care, which were maintained at follow-up. Trainees appreciated the interprofessional nature and “all teach, all learn” philosophy of the program, the inclusion of people with lived experience as teachers, and the discussion of real cases. The teaching team learned that trainees preferred a mix of chat and microphone participation. Initial hesitance due to perceived knowledge hierarchies was mitigated by the “all teach, all learn” philosophy and participant case presentations. This early evidence shows how adaptations of this successful continuing professional development model can benefit and engage trainees at an earlier stage.

Embracing immersive interprofessional training will equip trainees with the necessary skills and experiences to efficiently collaborate with their interprofessional colleagues. Interprofessional collaboration is especially relevant for adults with IDD, whose complex physical and mental health care needs often necessitate collaboration across health and social service fields [15, 19]. Providing trainees with educational opportunities to learn and gain competences in interprofessional collaboration will ensure that trainees will be better suited to manage a range of complex patient populations that require a team-based approach.

Having teachers with lived experience was an essential part of this program. Trainees valued and appreciated the unique perspectives that teachers with lived experience brought, helping consolidate learning which trainees indicated they would like to see included in other aspects of their education. Including individuals with lived experience of IDD has been increasingly utilized in health care education with positive results [20]. This study illustrates that including individuals with lived experience in an authentic and meaningful manner is impactful. As interprofessional education continues to expand and evolve, it becomes increasingly important to explore different ways that people with lived experience of disability and/or caregiving can contribute to teaching efforts.

Moving forward, the virtual ECHO teaching model can provide value to trainees and faculty alike as a tool to engage in interprofessional learning about complex patient populations, including adult IDD mental health. Including faculty from different departments and institutions in one virtual program addressed logistical accessibility for both faculty and trainees, who otherwise would not learn together and also ensured a sufficient number of trainees in the program. As health education increasingly addresses challenges faced by equity-deserving groups, the ECHO teaching model provides an interprofessional opportunity to engage trainees in an effective manner.

In terms of study limitations, all outcome measures were based on subjective experiences of trainees and not objective improvements in knowledge or skills and no patient-level outcomes were measured. Additionally, the experience of participants may not reflect trainees who did not participate in the study, who may also be less interested in working with this population. A more extensive evaluation with a longer follow-up in a larger cohort would be helpful in this regard. Increased emphasis on early interprofessional education for trainees regarding the mental health of adults with IDD is an important step to improving care. This virtual, inter-departmental, and inter-institutional model of learning may also have value for other low frequency, complex patient populations and should be further studied.

## Data Availability

Data is available upon reasonable request from the corresponding author.
